# Cardiac physiology and metabolic gene expression during late organogenesis among *F. heteroclitus* embryo families from crosses between pollution-sensitive and -resistant parents

**DOI:** 10.1186/s12862-022-01959-1

**Published:** 2022-01-07

**Authors:** Goran Bozinovic, Zuying Feng, Damian Shea, Marjorie F. Oleksiak

**Affiliations:** 1 Boz Life Science Research and Teaching Institute, San Diego, CA USA; 2 Department of Biological Sciences, North Carolina State University, Raleigh, NC USA; 3 Present Address: Division of Biological Sciences, University of California San Diego, San Diego, CA USA; 4 Present Address: Department of Marine Biology and Ecology, Rosenstiel School of Marine and Atmospheric Sciences, University of Miami, Miami, FL USA

**Keywords:** Gene expression, Metabolism, Development, Adaptation, Heart physiology

## Abstract

**Background:**

The teleost fish *Fundulus heteroclitus* inhabit estuaries heavily polluted with persistent and bioaccumulative chemicals. While embryos of parents from polluted sites are remarkably resistant to toxic sediment and develop normally, embryos of parents from relatively clean estuaries, when treated with polluted sediment extracts, are developmentally delayed, displaying deformities characteristic of pollution-induced embryotoxicity. To gain insight into parental effects on sensitive and resistant phenotypes during late organogenesis, we established sensitive, resistant, and crossed embryo families using five female and five male parents from relatively clean and predominantly PAH-polluted estuaries each, measured heart rates, and quantified individual embryo expression of 179 metabolic genes.

**Results:**

Pollution-induced embryotoxicity manifested as morphological deformities, significant developmental delays, and altered cardiac physiology was evident among sensitive embryos resulting from crosses between females and males from relatively clean estuaries. Significantly different heart rates among several geographically unrelated populations of sensitive, resistant, and crossed embryo families during late organogenesis and pre-hatching suggest site-specific adaptive cardiac physiology phenotypes relative to pollution exposure. Metabolic gene expression patterns (32 genes, 17.9%, at p < 0.05; 11 genes, 6.1%, at p < 0.01) among the embryo families indicate maternal pollutant deposition in the eggs and parental effects on gene expression and metabolic alterations.

**Conclusion:**

Heart rate differences among sensitive, resistant, and crossed embryos is a reliable phenotype for further explorations of adaptive mechanisms. While metabolic gene expression patterns among embryo families are suggestive of parental effects on several differentially expressed genes, a definitive adaptive signature and metabolic cost of resistant phenotypes is unclear and shows unexpected sensitive-resistant crossed embryo expression profiles. Our study highlights physiological and metabolic gene expression differences during a critical embryonic stage among pollution sensitive, resistant, and crossed embryo families, which may contribute to underlying resistance mechanisms observed in natural *F. heteroclitus* populations living in heavily contaminated estuaries.

**Supplementary Information:**

The online version contains supplementary material available at 10.1186/s12862-022-01959-1.

## Background

Understanding the mechanisms by which natural populations respond to stress, evolve adaptive genotypes, and diverge phenotypically has important implications for evolutionary biology and conservation ecology [1, 2]. Can populations evolve and adapt fast enough in response to human-caused strong selective pressures, including environmental pollution? The effects of pollution on populations often include increased mortality, decreased fecundity, and impaired physiological performances, but not all individuals respond the same: some are sensitive, while others adapt and become resistant [3–12]. Polluted stressful environments result in resource-deficient and energy-deficient phenotypes [13] and individual differences in stress response emerge through various gene–environment interactions [4, 8–10, 14].

**Fig. 1:**
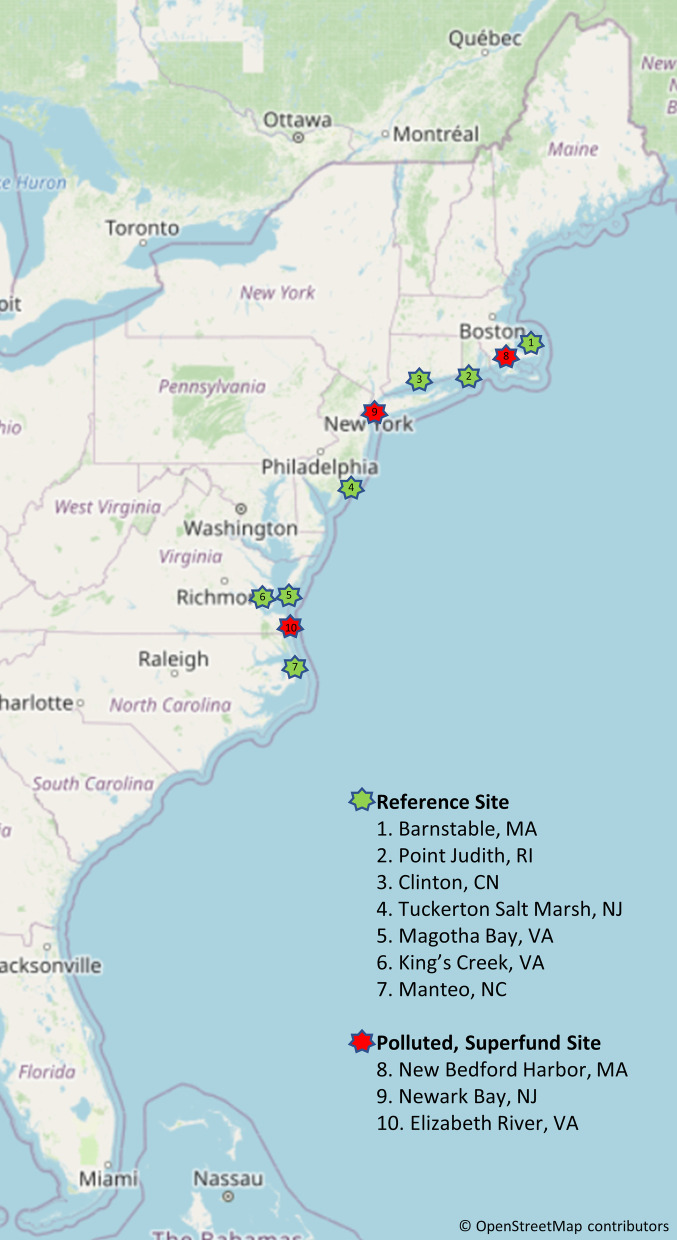
Sampling sites of PSD deployment and adult fish collection. Polluted and reference sampling sites of natural *F. heteroclitus* populations*.* Map data are obtained from OpenStreetMap^®^ (openstreetmap.org)

Atlantic killifish (*Fundulus heteroclitus*) is a unique ecological and evolutionary model with well-defined embryo development [15, 16], anatomy and physiology [17–20], and a reference genome sequence [21] to study rapidly evolved adaptative mechanisms to environmental pollution [3, 6, 20, 22, 23]. Since this small, nonmigratory fish has relatively small home ranges, natural selection can occur, but the effects of genetic drift are minimized, and locally specialized phenotypes may evolve quickly [24–26]. Large, genetically variable *F. heteroclitus* populations inhabit coastal marsh ecosystems along the East Coast of U.S., including urban, geographically unrelated estuaries heavily contaminated with persistent and bioaccumulative chemicals [27, 28]: New Bedford Harbor, MA, is mainly contaminated with polychlorinated biphenyls (PCBs) [29]; the lower Hudson River estuary in Newark Bay, NJ is mostly contaminated with PCBs, along with dioxins, polycyclic aromatic hydrocarbons (PAHs), and other toxic contaminants [30–32]; Elizabeth River, VA is contaminated with creosote, a complex mixture of PAHs [33, 34] (Fig. 1). Severe pollution has prompted the U.S. EPA to designate these three as Superfund sites and place them on the National Priority List [35]. EPA’s Superfund program is responsible for cleaning up U.S. most contaminated land and estuaries, and mitigating environmental emergencies, oil spills and natural disasters [36].

*Fundulus heteroclitus* is one of the few studied species in North America that has shown resistance to pollutants among both adults and embryos: while sediment extracts from such environments can be lethal to *F. heteroclitus* embryos from clean sites, embryos of parents from polluted sites are remarkably resistant [3, 7, 37–42]. Rapidly evolved tolerance phenotypes to environmental toxicants [11, 12, 43] have evolved independently multiple times [10, 11, 44], and each independent population can be associated with site-specific primary pollutants [5, 7, 8, 11, 20, 34]. While much is known about the molecular mechanisms of action for the major chemical pollutants to which they have adapted and about their transcriptome‐wide response to relevant chemical toxicants [3–6, 8–10], less is known about functionally important variation in gene expression underlying resistant phenotypes and signatures of adaptive divergence within and between populations.

An embryo is more susceptible to stress than an adult organism [45] due to surface area/volume ratio particularly in young fish, higher likelihood of juveniles having less fat and less capacity to store lipophilic compounds, greater uptake of environmental toxicants, immature detoxifying organs (kidney and liver) and naive immune system [46, 47]. Concentrations of persistent organic pollutants (POPs) measured in New Bedford Harbor, MA and Elizabeth River, VA are lethal to killifish embryos of parents collected from nearby reference estuaries [30, 31, 38, 41, 48, 49] and POP exposures can result in death, growth retardation, and quantifiable developmental deformities [38, 40, 50, 51]. Remarkably, the *F. heteroclitus* embryos from New Bedford Harbor, MA, Newark Bay, NJ, and Elizabeth River, VA do not exhibit such developmental deformities when treated with defined POP mixtures or sediment extracts from polluted estuaries [3, 30, 31, 52]. Physiological measures of stress often include heart rate variability [53] as the fluctuation of the length of heartbeat intervals represent the ability of the heart to respond to a variety of physiological and environmental stimuli. The heart is a target organ of PAH-induced developmental toxicity in *F. heteroclitus*. For example, embryonic exposure to weathered crude oil containing PAHs lead to impaired heart looping, atrioventricular conduction block, and cardiac ventricular conduction. We reported PAH-induced morphological and physiological cardiotoxicity in developing sensitive embryos during four critical developmental stages, while the severity of such effects was not observed in resistant embryo populations [7]. While embryo exposure to a simple PAH mixture results in cardiac deformities [3], exposure to individual PAHs alone at similar concentrations alters heart rates but does not induce morphological deformities [53]. Embryo‐larval cardiotoxicity of some low molecular weight PAHs may be mediated through disrupted regulation of intracellular potassium and calcium in cardiomyocytes and affect heart conductivity [54].

Since multiple loci are under selection in wild populations exposed to complex polluted environment, adaptive phenotypes are associated with a fitness cost may be complex; one type of environmental stress may increase the fitness cost as increased susceptibility to other stressors [55, 56], so traits that are adaptive for specific chemical contaminants may be disadvantageous under alternate conditions [34, 56–58]. Although all organs are fully differentiated and functional during *F. heteroclitus* late organogenesis (stage 31) [15], variation in expression patterns of protein-coding genes essential in metabolic pathways under strong selective pressure is likely, due to increased energy cost to protect an embryo from chemical pollution.

Gene expression variation plays an important role in adaptation, since molecular and physiological phenotypes can be affected by both genetic and environmental factors via *cis-* or *trans-* expression regulation and environmentally induced epigenetic regulation [59–64]. Therefore, gene expression level is considered an intermediate phenotype leading to new adaptive traits [65, 66]. Signatures of selection in wild populations indicate a complex adaptive phenotype, such as adaptation to chemicals with different modes of action [5, 7, 8, 11, 20, 34] and adaptation to other stressors in altered habitats (e.g., hypoxia, altered pathogen community) [37, 67]. Phenotypic changes during development are important for adaptation to heavily polluted environments [3, 5, 7]. Some might be environmentally induced, affected by maternal deposition, while others may be due to long-term evolved changes inherited across generations. Population hybridization is an important factor in genomic evolution and speciation resulting from the merger of two sets of divergent genomes [68, 69] and can have pronounced fitness consequences. However, knowledge regarding how hybridization affects gene expression and gene regulation is limited. Sudden change in gene expression following the mixing of two dissimilar parental genomes each with individual sets of transcription factors and chromatin profiles [70–72] could be used to assess parental contribution to the gene expression levels either temporally or spatially during hybrid development. One approach to identify cross-generational changes is to compare embryo crosses between parental fish from both sensitive and resistant populations focusing on expression of genes coding for major enzymes in metabolic pathways. Inherited embryo phenotypes resulting from CP and PC crosses (the first letter denotes a female parent, and the second denotes a male parent; “C” refers to a relatively “Clean” parental origin site, and “P” refers to a “Polluted” parental origin site) are likely to be intermediate between embryos from sensitive (CC) and resistant (PP) populations. Using sensitive and resistant embryos’ differential gene expression as references, CP and PC expression profiles could suggest maternally contributed pollutant exposure in the egg (evident as a PC-unique gene expression pattern) and parental effects on gene expression, providing a unique viewpoint of the pollution adaptive phenotype.

To explore environmentally induced and evolved phenotypic changes in resistant *F. heteroclitus* populations, we first assessed sampling sites for PAHs and PCBs chemical content using passive sampling devices [73, 74]; we evaluated embryotoxicity during late organogenesis among pollution-sensitive embryos from King's Creek, VA, a commonly used reference site in several studies reporting pollution-induced sensitive and resistant phenotypes of *F. heteroclitus* populations exposed to pollution (Fig. 1) [3, 34, 58], by exposing them to predominantly PAH-polluted sediment extracts from Elizabeth River, VA. We then established crosses between two sets of sensitive/resistant *F. heteroclitus* populations to assess heart rate differences between embryo families: (1) Southern pollution-resistant Elizabeth River fish and pollution-sensitive fish from a nearby reference population in Magotha Bay, VA, and (2) Northern pollution-resistant Newark Bay fish and pollution-sensitive fish from a nearby reference population in Tuckerton, NJ (Fig. 1). We assessed the heart rate differences between sensitive, resistant, and crossed embryos families from both sets and quantified the expression of 179 metabolic genes among individual embryos of four family structures from polluted Elizabeth River and reference Magotha Bay fish crosses. These analyses give insights into parental effects on pollution sensitive and resistant phenotypes among developing embryos from natural populations.

## Results

### Sampling sites chemical analysis

Assessment of on-site chemical exposures using passive sampling devices (PSDs) revealed highest concentration of total PAHs, including pyrene, phenanthrene, and fluoranthene, in Elizabeth River, VA sediment samples (5630 ng/sample) relative to 11.6 ng/sample identified at the Magotha Bay, VA reference site (Table 1). The concentrations of total PAHs at Elizabeth River site were 487-fold and 195-fold greater compared to the two reference sites, Magotha Bay, VA and Manteo, NC, respectively (Table 1). A similar pattern was observed comparing northern polluted and reference sites: the concentrations of total PAHs were significantly higher in PSDs deployed at Newark Bay, NJ relative to nearby southern (3.5-fold; Tuckerton, NJ) and northern (5.76-fold; Pt. Judith, RI) reference sites (Table 1). The highest total concentrations of PCBs were measured in New Bedford harbor, MA (660 ng/samples), more that tenfold higher than Newark Bay, NJ (54 ng/sample), and almost 100-fold more than in Elizabeth River, VA (5.2 ng/sample). All reference sites had relatively low concentrations of total PCBs (< 3.4 ng/sample).

**Table 1:** PSD site data for average total PAHs, three representative PAH compounds, and total PCBs measured per one PSD

Study sites	Site GPS coordinates	Maximum concentrations measured per PSD (ng/sample)				
		Total PAHs	Pyrene	Phenanthrene	Fluoranthene	Total PCBs
Magotha Bay, VA	37° 10.6′ N 75° 56.5′ W	11.55	< 0.1	1.57	1.02	1.9
**Elizabeth River, VA**	**36° 48.5′ N 76° 17.7**′ **W**	**5630**	**692**	**230**	**2350**	**6.2**
Manteo, NC	35° 53.8′ N 75° 36.9′W	28.8	1.23	1.49	0.97	1.3
Clinton, CT	41° 15.3′ N 72° 32.8′ W	135	11.7	20.12	30.3	3.7
**Newark Bay, NJ**	**40° 41.2′ N 74° 6.7**′ **W**	**224**	**20.4**	**17.3**	**29.4**	**54**
Tuckerton, NJ	39° 32.2′ N 74° 19.4′ W	63.7	5.79	6.06	19.2	2.6
Sandwich, MA	41° 44.0′ N 70° 23.0′ W	37	< 0.1	6.04	0.6	4.1
**New Bedford Harbor, MA**	**41° 39.9′ N 70° 54.9**′ **W**	**96.2**	**9.78**	**7.48**	**13.4**	**660**
Pt. Judith, RI	41° 21.7′ N 71° 28.9′W	38.9	2.4	5.14	3.07	2.1

Six PSDs were deployed at each site. Polluted sites are in bold

### Effects of polluted Elizabeth River sediment extracts on sensitive embryo mortality, morphology, developmental delays, and heart rate

**Fig. 2:**
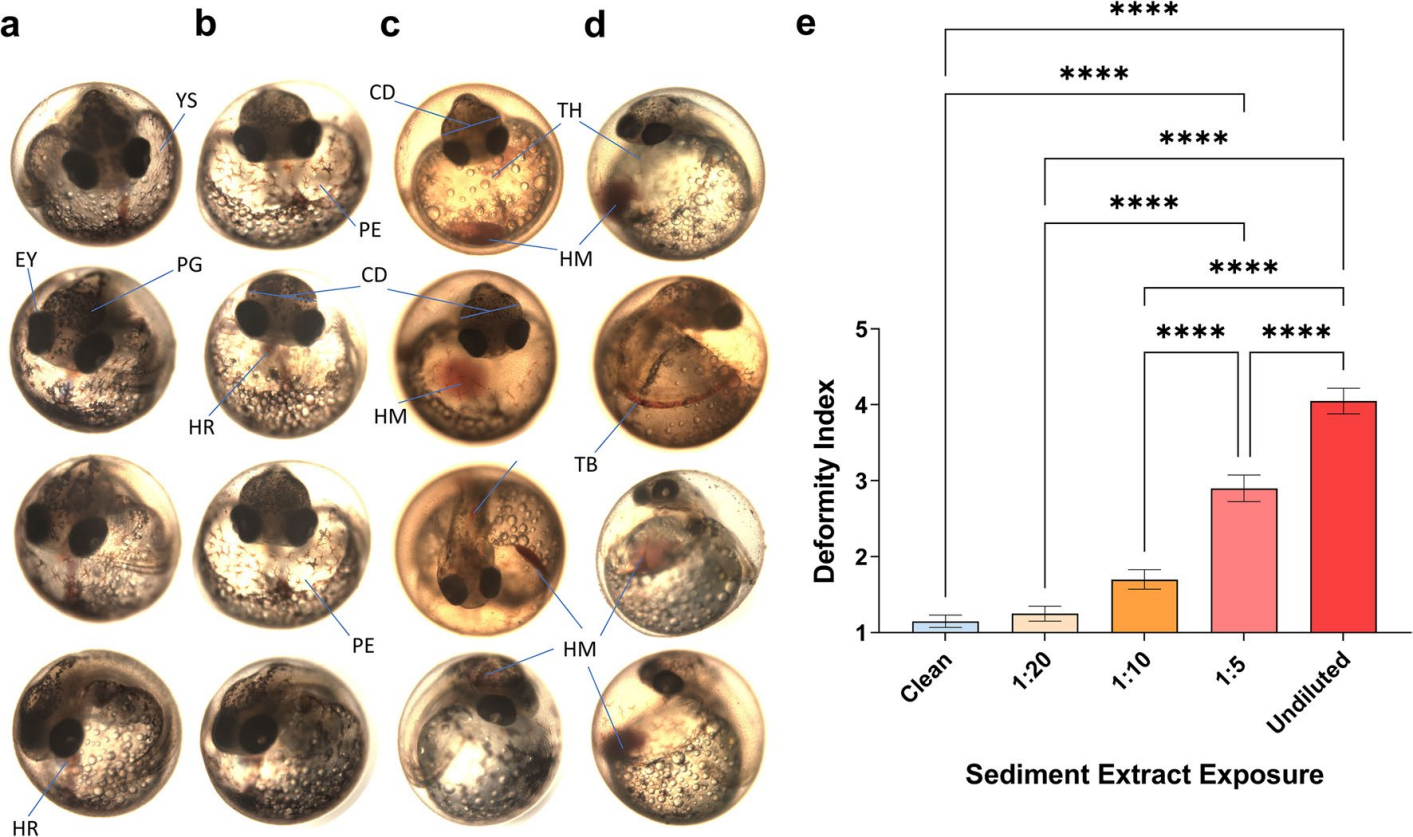
Effects of PAHs exposure on sensitive *F. heteroclitus* embryo’s morphological development at stage 31. Representative photos show embryos with** a** normal development as well as** b** mild,** c** moderate, and **d** severe-to-extreme deformities. CD, cranial distance; EY, eye; HM, hemorrhage; HR, heart; PE, pericardial edema; PG, pigment; TB, tail bleed; TH, tube heart; YS, yolk sac.** e** Embryo deformity is significantly more severe in higher concentrations of Elizabeth River sediment extracts (n = 20 per exposure condition). Deformity of each individual embryo was scored based on the protocol specified in the Methods. ****p < 0.0001

**Fig. 3:**
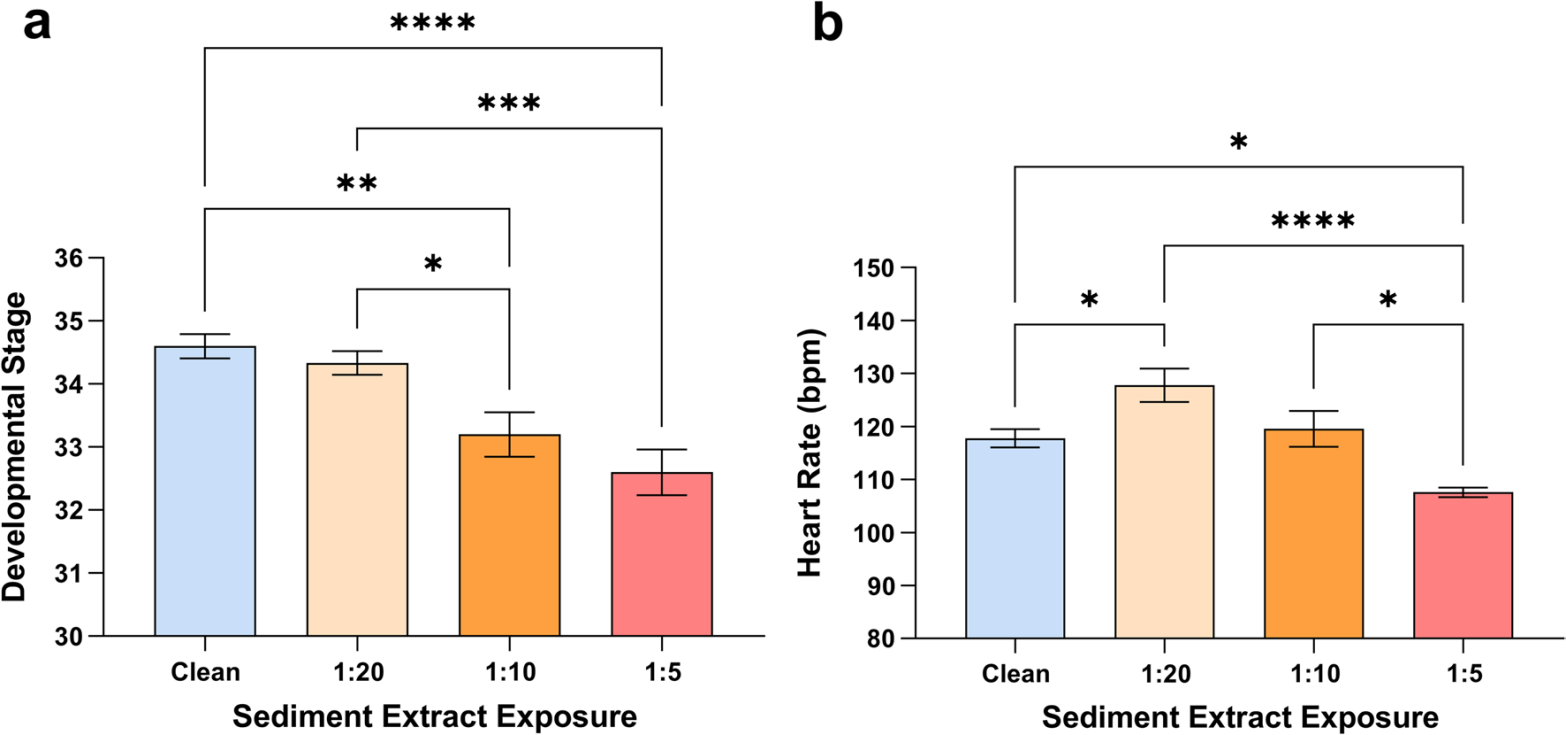
Exposure to diluted sediment extracts cause developmental delays, heart rate changes, and deformities in sensitive embryos from King’s Creek, VA. **a** Developmental delay of sensitive embryos exposed to differently diluted Elizabeth River sediment extracts (n = 15 per exposure condition). Embryos exposed to 1:10 and 1:5 diluted sediment extracts showed delayed development. Stages were determined after 10 days and 16 h post-fertilization.** b** Heart rate differences among sensitive embryos exposed to differently diluted Elizabeth River sediment extracts (n = 10 per exposure condition). Heart rates were determined at stage 31. *p < 0.05; **p < 0.01; ***p < 0.001; ****p < 0.0001

Significant adverse morphological effects were observed among King’s Creek sensitive embryos exposed to polluted undiluted and serially diluted sediment extracts, with embryo treatment groups responding in a dose–response manner (Fig. 2). The severe-to-extreme malformations characterized by elongated (tube) hearts, pericardial edema, tail vessel hemorrhaging, cranial deformities, and a loss of pigment were noted among 75% embryos (n = 20) exposed to undiluted sediment extracts, with an average deformity index (DI) score of 4.4 (scale 1–5; 1 = normal, 5 = extremely deformed). Fifteen percent of embryos exhibited severe abnormalities, while 55% were moderately deformed among embryos exposed to 1:5 diluted extracts (DI = 2.9) (Fig. 2b). Embryos exposed to 1:10 sediment extract dilution resulted in 60% of embryos mildly deformed (DI = 1.7), while exposure to 1:20 diluted extracts resulted in no significant embryo abnormalities (DI = 1.25) relative to the normally developed control group (DI = 1.15) (Fig. 2b). None of the sensitive embryos exposed to undiluted sediments hatched and developed past stage 35 due to severe developmental malformations and irreversibly compromised cardiac function. Notably, highest-dosed embryos (1:5 dilution) were developmentally delayed, with > 70% embryos between stages 31–33, while > 90% of the control embryos reached stage 35 as expected (Fig. 3a).

Average heart rate among embryos exposed to 1:20-diluted PAH-contaminated sediments during the pre-hatching stage [15] was 127.8 bpm, significantly faster than heart rates of embryos developing in clean water (117.8 bpm; p < 0.05). Heart rate of embryos developing in 1:5 diluted extracts (107.6 bpm) was significantly slower than other treatment groups (p < 0.05), while 1:10 treatment embryos (119.6 bpm) did not differ from the control (Fig. 3b). Cardiac function among extremely deformed sensitive embryos exposed to undiluted extracts was severely compromised by stage 35 (below 60 bpm) and resulted in embryos’ death.

### Heart rates and morphology of sensitive (Magotha Bay, VA), resistant (Elizabeth River, VA), and crossed embryos developing in clean water

**Fig. 4:**
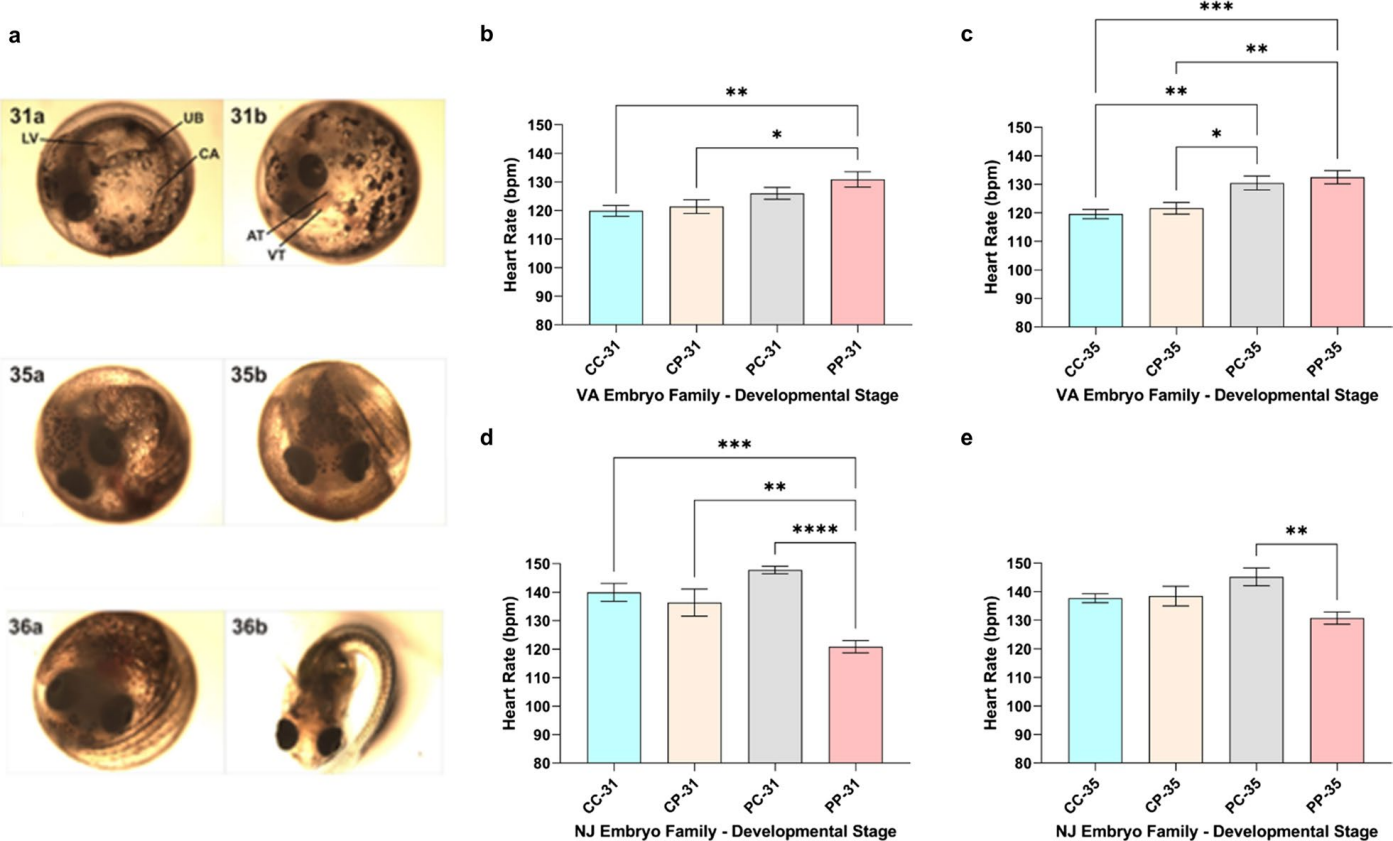
Embryo morphology, heart rates of sensitive, resistant, and crossed embryos differ by developmental stages. **a** *F. heteroclitus* embryos at developmental stages 31, 35, and 36. LV: liver; UB: urinary bladder; CA: cartilage; AT: atrium; VT: ventricle. Stage 31: Heart chambers are differentiated under the cephalic region left atrium and the right ventricle rostrally. The liver circulation through the sinusoids can be observed. The bilobed urinary bladder contains visible precipitate. Stage 35: Pre-Hatching. Continuous elongation of the head causes further separation of the ventral head region from the yolk. The eyes are large and move frequently. The liver increases in size and obscures the view of the gall bladder. The tail can reach beyond the caudal border of the eye. The tip of the tail can reach beyond the otic vesicle and the embryo often rotates within the chorion. Stage 36: Hatching. The more frequent opening of the lower jaw indicates the beginning of the hatching, during which the hatching enzyme is released from unicellular glands in mucosa of buccal and pharyngeal regions. The chorion eventually lyses and the embryo breaks free tail-first. The tail straightens immediately upon hatching, and the embryo can swim within the hatching medium. **b**, **c** Heart rates of sensitive, resistant, and crossed embryos (n = 20 per family structure) whose parents were collected at Magotha Bay, VA and Elizabeth River, VA. **d**, **e** Heart rates differences among sensitive, resistant, and crossed embryos (n = 14 per family structure) whose parents were collected at Tuckerton, NJ and Newark Bay, NJ. Heart rates were determined at stage 31 (**b**, **d**) and 35 (**c**, **e**). *p < 0.05; **p < 0.01; ***p < 0.001; ****p < 0.0001

There were no observable morphological abnormalities resulting in developmental delays among four embryos families developing in clean water. During the late organogenesis stage, heart rates among sensitive (119.9 bpm), CP (121.4 bpm) and PC (126 bpm) embryos were not statistically different (Fig. 4b). The PP resistant embryos’ heart rates (130 bpm) were statistically faster than CC embryos’ (p < 0.01). This trend continued during pre-hatching stage 35, with the sensitive embryos’ mean heart rate of 119.6 bpm, which did not differ significantly from CP embryos’ mean heart rate (121.6 bpm). However, the average heart rates of PC (130.5 bpm) and PP (132.6 bpm) embryos were significantly higher than those of CC and CP embryos (Fig. 4c, p < 0.05).

### Heart rates and morphology among sensitive (Tuckerton, NJ), resistant (Newark Bay, NJ), and crossed embryos

While there were no observable morphological abnormalities among embryo families resulting in delayed development, our results indicate developmental stage-dependent differences in heart rates between sensitive and resistant embryos. Significantly slower heart rates (p < 0.01) were measured among PP embryos at stage 31 relative to other embryo groups (Fig. 4d). The mean heart rate among CC embryos at stage 31 was 139.9 bpm, while the mean heart rate among PP embryos at the same developmental stage was 120.9 bpm (Fig. 4d). The mean heart rate of CC embryos at stage 35 was 137.7 bpm, while the PP mean embryo heart rate was 130.8 bpm (Fig. 4e). Heart rate differences were more significant during stage 31 than 35, with most of the PP embryos having elevated heart rates during the latter pre-hatching stage.

### Gene expression

**Fig. 5:**
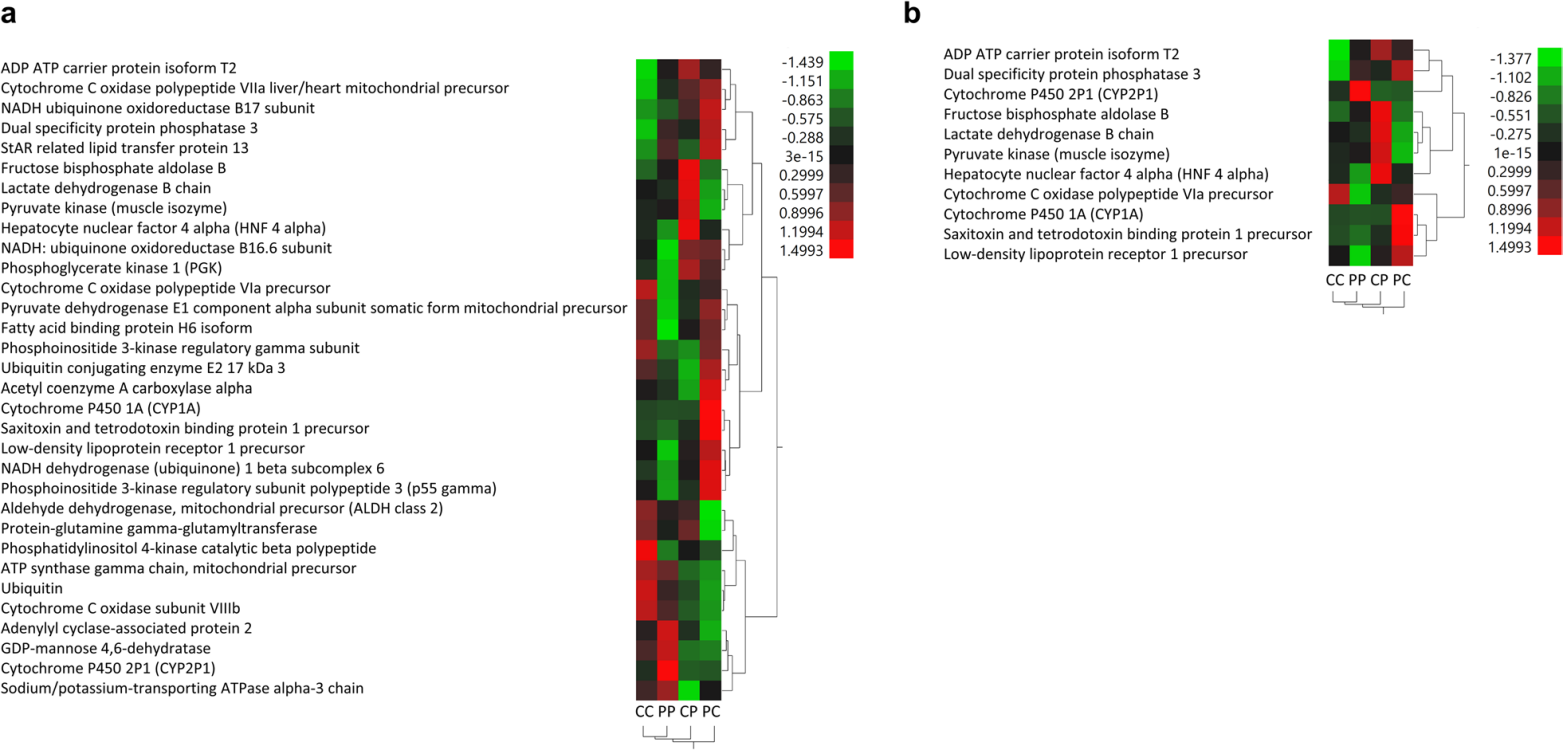
Hierarchical clustering of genes whose expression level significantly differ among four family structures. **a** Heatmap of 32 genes whose expression was significantly different (p < 0.05). **b** Heatmap of 11 genes whose expression was significantly different (p < 0.01). Red indicates high expression levels, while green indicates low expressions levels

**Fig. 6:**
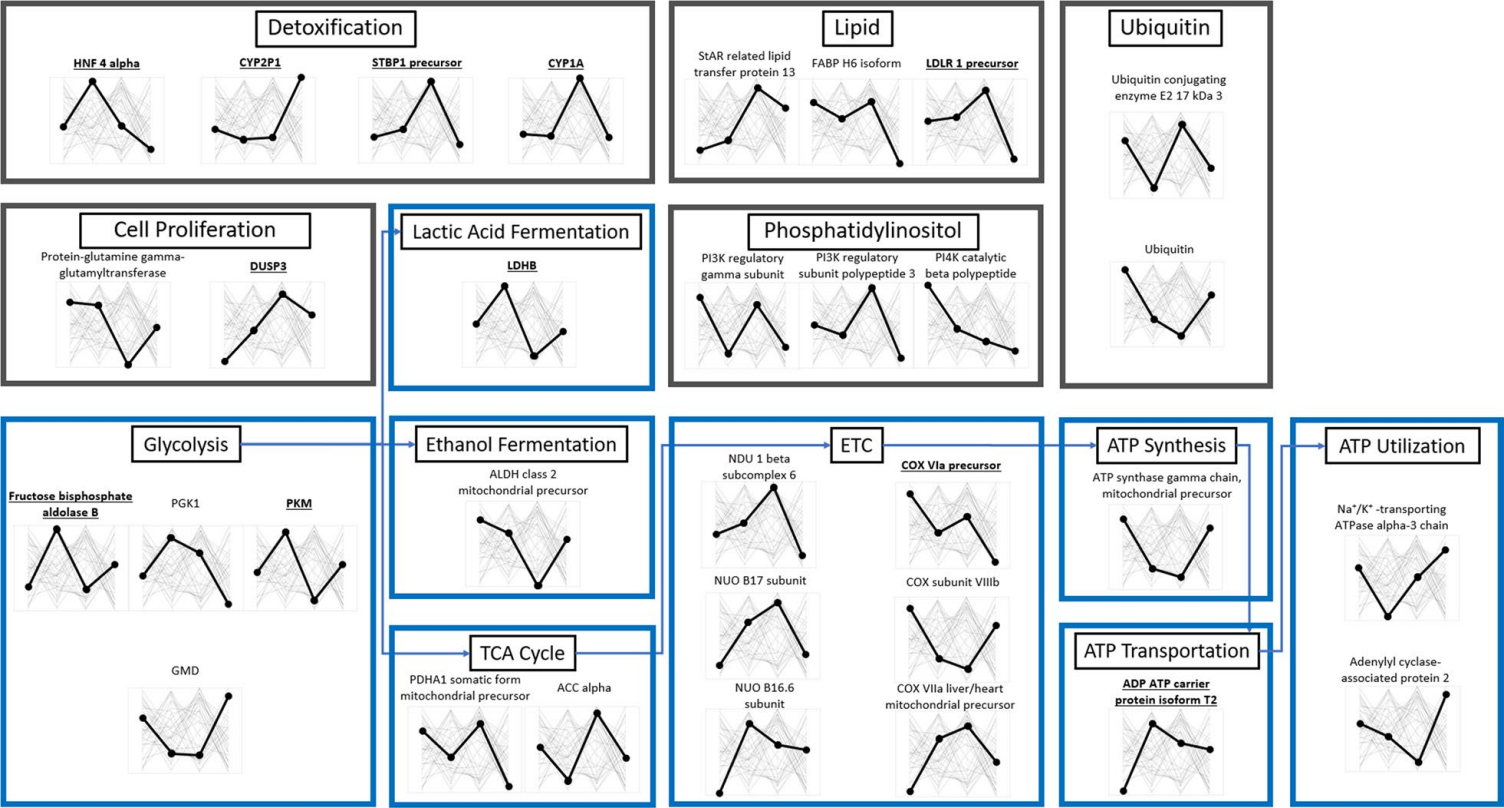
Pathway analysis of differentially expressed genes (p < 0.05) among four embryo family structures. From left to right: CC, CP, PC, and PP. The bolded lines show the expression trends of the gene above the line across the four family structures, while other lines represent the expression of other significant genes. Significant genes at p < 0.01 are bolded and underlined

**Fig. 7:**
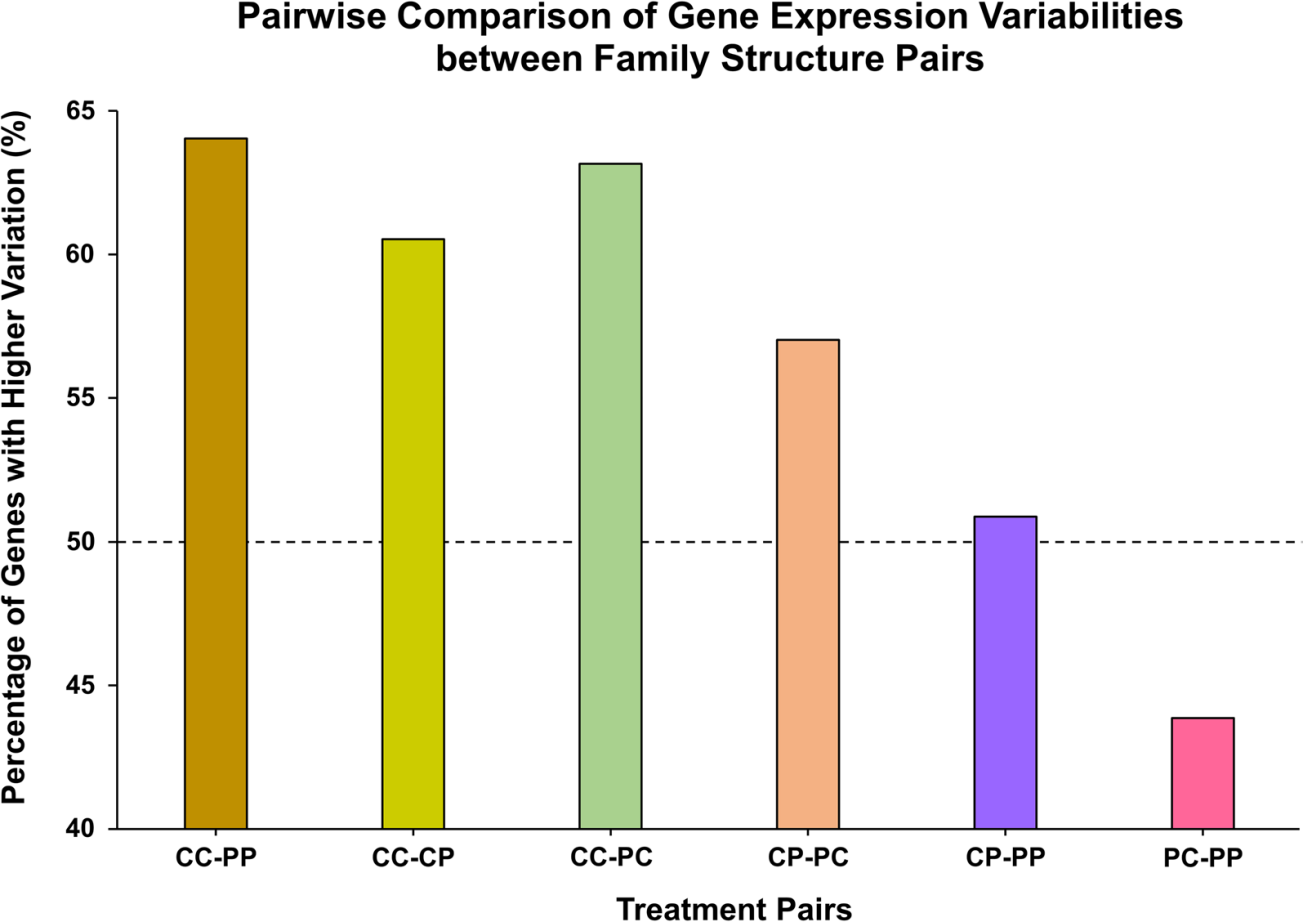
Pairwise comparisons of gene expression variability between family structure pairs. The family structure on the left side of each pair is compared to the one on the right side for expression variability, and the results are displayed as percentages of more variable genes among all genes. CC group has the most genes with higher expression variations, while PC has the fewest

The expression of 17.9% (32/179) metabolic genes was statistically different per embryo population (p < 0.05) (Fig. 5a), and 6.1% (11/179) had statistically different expression patterns at p < 0.01 (Fig. 5b). Functional analysis and expression patterns of the 32 genes with significantly altered expression are summarized in Fig. 6: majority (18) are implicated in cellular respiration pathways, while others are associated with toxic substance responses (4), lipid metabolism (3), phosphatidylinositol phosphorylation (3), and ubiquitination (2). Expectedly, of the 114 significantly variable metabolic genes (p < 0.01), CC embryos had the highest number of variable genes among the four family structures. Sixty-three percent of these genes have significantly lower expression variation in the PP embryos compared to CC embryos (Fig. 7, p < 0.01). Both crossed embryo groups had significantly lower number of variable genes compared to CC embryos (CP = 61%, PC = 63%), while 57% of 114 genes in CP embryos were more variable than in PC embryos.

## Discussion

Our previous work with *F. heteroclitus* embryos provided anatomical guidelines and statistical references for the temporal dynamics of developmental gene expression during all stages of vertebrate development [15]. By assessing sensitivity and resistance of multiple *F. heteroclitus* natural embryo populations, we showed that statistically significant changes in gene expression of relatively few genes contribute to large morphological differences in sensitive and resistant embryos exposed to PAH pollutants [3]. Such differences may be masked by developmental canalization, and biologically important phenotypes between sensitive and resistant embryos may only manifest with exposure and are dependent on gene-environment interactions.

### PSD-based chemical analysis confirms site-specific predominant contaminants at Superfund sites

Chemical analysis of the embryos’ parental environment utilizing deployed PSDs at the testing sites shows marked differences in PAH concentrations in sediment extracts between southern (Magotha Bay, VA and Manteo, NC) and northern (Pt. Judith, RI and Tuckerton, NJ) reference sites compared to polluted (Elizabeth River, VA and Newark Bay, NJ) sites (Fig. 1). The Elizabeth River and Newark Bay sites are heavily contaminated with numerous low- and high-molecular weight PAHs, which have been shown to elicit toxicological effects during *F. heteroclitus* embryogenesis [37, 51, 75]. The highest concentration of total PAHs was measured at the Elizabeth River site, confirming the significant differences in contamination and potential levels of exposure to site biota. The concentration of total PAHs at the contaminated superfund site is strikingly 487-fold and 195-fold greater at Elizabeth River site compared to the two reference sites, respectively.

While Elizabeth River, VA is the site most polluted with PAHs, concentrations of PCBs are relatively low, and much closer to the range with all reference sites. Although New Bedford Harbor, MA has the highest concentrations of PCBs measured among three polluted sites, Newark Bay, NJ has more than ten-fold higher total PCB contamination than Elizabeth River, VA, while total PAH concentration in Elizabeth River, VA is more than 25-fold greater than in Newark Bay, NJ. We previously reported 32.5-fold increase in PAH concentration in Elizabeth River, VA relative to Newark Bay, NJ [5]. Although there are measurable concentrations of representative low molecular weight PAHs in several reference sites, the total PAH concentration differences are at least 40-fold in magnitude lower than that in Elizabeth River, VA, and about 60% less than in Newark Bay, NJ. When comparing PCB total concentrations in Newark Bay, NJ and relevant reference sites, Newark Bay has > 14-fold high concentration of PCBs.

All three Superfund sites are marked by both PAH and PCB contamination; considering predominant POP type and PAH molecular weights at each site, southern Elizabeth River site is characterized as predominantly PAH-polluted, and northern Newark Bay as PCB-polluted. Reference sites relative to each Superfund site were selected as less polluted and located in the non-urban, often ecologically protected less contaminated locations, close enough so the population differences are due to pollution alone. Our PSD data confirms the reference sites are much cleaner than polluted superfund sites.

Since PSDs mimic biological membrane composition [76], the exposure levels at the contaminated sites and potential acute and chronic toxicity affecting natural populations’ fitness are alarming. In contrast, we detected low concentrations of two low-molecular weight PAHs (phenanthrene and fluoranthene) in reference site water, indicating that the parents of embryos sensitive to PAHs live in a relatively clean environment as compared to fish from the highly PAH-polluted Elizabeth River and Newark Bay sites. PSDs passively accumulate the dissolved and bioavailable fraction of organic chemicals and are used to estimate time-weighted-average exposure to PAHs. These membranes are not susceptible to biological variation affecting bioaccumulation in living organisms and correlate well with PAH accumulation in caged mussels [74]. Chemical analysis of sampling devices confirms the significant site-specific differences in exposures to primary POPs and selective pressure to adult and embryo fish populations at relatively clean and contaminated sites.

### Embryos of parents from reference sites exposed to PAH-polluted sediment extracts display morphological deformities, developmental delays, and altered cardiac physiology

While the chorion membrane, due to its multi-protein layered structure [77] provides a barrier to estuarine toxicants during embryo development, the laboratory exposures indicate that significant concentrations of toxicants penetrate the chorion and adversely affect embryo development [7]. Responses of sensitive embryos to higher concentrations of PAHs reflect their ability to detoxify and eliminate PAHs during organogenesis (pre-stage 31) and post-hatching (after stage 35). By stage 31, the liver, which expresses CYP450 family enzymes with a critical role in PAH detoxification [78], is functional. Tube hearts, pericardial swelling, and reduced circulation were reported among the sensitive embryos exposed to Elizabeth River sediment [3, 37, 75, 79]. Similar effects were noted in embryos of parents recently caught in the field and embryos whose parents were raised in the lab. However, embryos of parents from polluted sites were significantly more tolerant to PAH-contaminated sediments relative to formation of cardiac abnormalities [41, 80].

In this study, we exposed embryos of parents from King’s Creek reference site to diluted, polluted sediment extract to verify embryo sensitivity in a dose–response manner and quantify adverse effects of Elizabeth River sediment before assessing morphological and physiological outcomes. We observed characteristic hallmarks or embryotoxicity, including altered cardiac morphology and physiology among sensitive embryo populations exposed to sediment extracts from Elizabeth River, VA (Fig. 2a). Expectedly, whole-sediment extract exposures to sensitive embryos resulted in extreme-to-severe morphological abnormalities and irreversible cardiac malformations characterized by formation of “tube hearts”, ultimately resulting in embryos’ death in ovum. The most pronounced heart abnormalities (“tube hearts”, pericardial edema, cranial and tail hemorrhaging) were noted among the highest-dosed embryos (Fig. 2), with the embryo treatment groups responding in a dose–response manner. Average heart rate was significantly slower among the embryos exposed to highest sediment extract concentration (1:5 dilution), which is consistent with the pattern reported previously [7], while the 1:10 dilution treatment group did not differ from the control. Interestingly, the embryos exposed to lowest sediment concentration (1:20 dilution) had, on average, significantly faster heart rates relative to control (Fig. 3b), which was similar to the results among the PP and PC embryo heart rates developing in clean water (Fig. 4b). Plausibly, embryo hearts beat faster due to lower PAH-exposures, while higher concentrations and long-term chronic exposures result in the slower heart rate and delayed development.

Although there were no statistical differences in the mortality rates among the sensitive embryos exposed to diluted sediment extracts from Elizabeth River Superfund site, the development of the highest-dosed embryos (1:5 dilution) was significantly delayed (Fig. 3a) and marked by dose–response characteristic hallmarks of PAH-induced embryotoxicity among two higher-dosed embryo treatment groups exposed to polluted sediment extracts. Mild-to-moderate structural heart abnormalities marked by pericardial edema and heart ventricle “thinning” were observed in the highest-dosed control embryo treatment group (1:5 dilution). Other observed effects include loss of pigmentation, shorter eye distance, loss of distinct craniofacial markers, systemic and tail hemorrhaging, resulting in decreased systemic circulation capacity. Our recent report shows that maternal deposition significantly contributes for PAHs concentrations exposure to developing sensitive and resistant embryos; resistant embryos from Elizabeth River site have significantly higher yolk concentration of PAH site-representative pollutants than reference sensitive embryos [7] in five critical developmental states, ranging from unfertilized egg to per-hatching.

Our goal here was to confirm sensitivity of embryo families based on observed phenotypes before we assess heart rate and gene expression differences between embryo family structures. However, the observed adverse effects on embryos from parents captured at reference sites due to exposures to PAH-contaminated sediments is consistent with reported results based on PAH exposures [37, 38, 51, 75, 79].

### Southern and northern sensitive, resistant, and crossed embryos display site-specific cardiac physiology

We utilized embryos of parents from southern (VA) and northern (NJ) reference and polluted sites and embryos of pollution sensitive and resistant parental crosses to assess differences in heart rates. While sensitive embryos exposed to polluted sediments, including sensitive King’s Creek embryos exposed to Elizabeth River polluted sediment extracts (Fig. 2) show phenotypic alterations characteristic of PAH-induced embryotoxicity [3, 7, 38, 39], no such effects nor developmental delays were observed among embryo families developing in clean water (Fig. 4a).

While rearing all four embryo families—sensitive, resistant, and crossed (CP and PC) embryos in clean conditions could be considered a “reverse stress” to resistant and crossed embryos by not developing is more “familiar” polluted extracts, we used a single treatment condition to minimize exposure variables. Moreover, our recent study results using chemically characterized, relatively clean and PAH-polluted sediments extract to rear sensitive and resistant embryos reveals no significant phenotypic differences in embryo morphology and developmental delays, between sensitive and resistant embryos developing in clean sediment extracts during both late organogenesis stage 31 and pre-hatching stage 35 [5, 7].

We previously reported the phenotypic differences between sensitive and resistant embryos developing in either clean or polluted sediment extract: characteristic hallmarks of PAH-induced embryotoxicity, including severe-extreme morphological alterations and impaired cardiac function evident among sensitive embryos, were not observed among resistant embryos developing in either clean or PAH-polluted sediment extracts [5, 7]. Notably, significantly higher heart rates among resistant embryos reared in clean water relative to sensitive embryos in both Elizabeth River and Newark Bay killifish populations, with Elizabeth River embryos having higher heart rates of the two [5], suggested differential, site-specific cardiac physiological response to clean environment among resistant embryo populations [5]. Hence, by crossing sensitive and resistant parents, we assessed the parental effect on the heart rate phenotype as a marker of pollution-induced, either via maternal pollution deposition or direct exposure, compromised cardiac physiology and metabolism, testing possible site-specific alterations in cardiac physiology during late organogenesis and pre-hatching among both sensitive and resistant *F. heteroclitus* populations from multiple reference and polluted sites. Four groups of ten embryos in both southern and northern population sets were assessed for the differences in heart rate during late organogenesis (stage 31) and pre-hatching (stage 35), and time-to-stage, as an indicator of developmental delays. A beating heart is formed, with both chambers completely differentiated and in full view by stage 31, and the heart rate can be accurately measured from that stage on [15]. Significant heart rate differences were measured in resistant embryos of parents from Elizabeth River, VA and Newark Bay, NJ relative to their controls at stage 31, but the observed trends differed: heart rates of Elizabeth River embryos were significantly higher than those of Magotha Bay embryos, while heart rates of Newark Bay embryos were significantly slower than those of Tuckerton embryos during stage 31 (Fig. 4b, d). The same pattern was observed during stage 35 (Fig. 4c, e).

For the southern VA embryos, the average heart rate among sensitive embryo populations did not differ significantly from crossed embryos of sensitive mothers and resistant fathers (CP). However, the average heart rates of both embryos of resistant mother/sensitive father (PC; 130.5 bpm) and resistant embryos (PP; 132.6 bpm) at the pre-hatching stage were significantly higher relative to CC and CP embryos (Fig. 4c, p < 0.05), suggesting metabolic alterations associated with increased heart rate and pronounced maternal effect. Elizabeth River embryos’ heart rates show a clear maternal correlation because CP embryos heart rate values are close to CC embryo hearts rates, whereas PC embryos display resistant (PP)-like heart rate values. By contrast, this pattern is absent among northern NJ embryo families, as PCs have significantly higher heart rate values relative to resistant (PP) embryos. Fish generally metabolize PCBs, the major pollutant at Newark Bay, NJ, more slowly than PAHs [81], and the maternal deposition effect on embryotoxicity could play a greater role than within PAH-exposed populations. Considering the acclimation of northern embryos to colder temperatures, differences in annual temperature fluctuations, chemical categorization of polluted sites as compound-specific aspect of mechanisms of action that influence chemical-specific toxicity and potential adaptive mechanisms [33, 34, 42], and our PSD characterization of predominant pollutants at each site (PAHs at Elizabeth River, VA and PCBs at Newark Bay, NJ), our results are suggestive of geographical and site-specific factors. Since there is a significant temporal and spatial variation in sediment contamination at the polluted sites [82, 83], a chemical characterization of sensitive, resistant, and crossed embryos developing in clean water at stage 31 and 35 would further discern the effect of maternal pollutant transfer vs. maternal effect on gene expression on heart rates.

### Metabolic gene expression of sensitive, resistant, and crossed embryos

Per hierarchical clustering of 32 differentially expressed genes (Fig. 5a, p < 0.05; Fig. 5b, p < 0.01), gene expression between CC and PP embryos are more similar than CP and PC embryos. While two genes (p < 0.05) in CP and PC embryos show intermediate phenotype relative to sensitive and resistant embryos, most genes show no clear pattern indicative of parental role in embryo gene expression. Functional analysis of 32 significant genes (p < 0.05) highlights six main processes: toxic substance response, lipid metabolism, phosphatidylinositol phosphorylation, protein ubiquitination, cell proliferation, and cellular respiration and ATP utilization (Fig. 6). The significant changes in gene expression are not surprising, but the expression patterns per embryo family are (Fig. 6). Toxic substance response (four genes) and lipid metabolism (three genes) are expected because of multigenerational exposure of *F. heteroclitus* to high concentrations of environmental toxicants, primarily PAHs that partition into lipid-rich tissues [84]. *F. heteroclitus* mothers from polluted estuary lower their body PAH body burdens via spawning by depositing toxicants in fatty-rich egg yolk content, a primary nutrient source for the developing embryos; once the fats are utilized by the developing embryo, PAH-induced embryotoxicity is likely to increase [85].

The alterations of phosphatidylinositol phosphorylation (three genes) and ubiquitination (two genes) are interesting, since both are critical cellular regulation pathways implicated in PAH-induced carcinogenicity [86–90]. Considering the observed developmental delays in sensitive embryos exposed to polluted sediments (Fig. 3a) and possible PAH-activated tumor-suppression mechanisms, alteration in expression of two genes linked to cell proliferation is not surprising. Likewise, since cellular respiration is a primary source of converting nutrients to ATP, both substrate-level phosphorylation (glycolysis—three genes) and cellular respiration pathways (TCA cycle—two genes, lactic acid and ethanol fermentation—one gene each, ETC—six genes, and ATP synthesis fate and transportation—three genes) are significantly affected.

HNF 4 alpha codes for nuclear receptor critical for expression of liver genes and controls sexually dimorphic expression of CYP family genes. In *F. heteroclitus* this gene plays a role in salinity regulation in a population-dependent manner [22] and is upregulated within a New Bedford Harbor Superfund site PCB-exposed population [4]. HNF 4 alpha is downregulated in resistant embryos but upregulated in CP embryos, suggesting paternal effect. Notably, the expression of CYP1A, encoding a major enzyme in xenobiotic Phase I metabolism and detoxification/metabolic activation of PAHs, is significantly upregulated in PC embryos. CYP1A is refractory to PAH induction in resistant embryos exposed to polluted PAH sediment extracts and among the *F. heteroclitus* resistant adults and embryos that live in highly polluted sites [37, 91], which is one of the proposed mechanisms of resistance observed in resistant *F. heteroclitus* populations. The PAH-induced cardiac morphology abnormalities—pericardial edema and malformed tube heart—that often result in embryo mortality, were reported among *F. heteroclitus* embryos of parents from reference sites exposed to CYP1A-inducers in the lab [37]. Fish metabolize PAHs via induction of CYP1A relatively quickly, but metabolic activation leads to formation of secondary active metabolites that are often more toxic than the parent compound [81, 90]. The relatively low expression levels of CYP1A in sensitive, resistant, and CP embryos are thus expected, while the upregulation of CYP1A among PC embryos is likely due to maternal PAH egg deposition. Plausibly, maternal contribution of PAHs-resistant genotype alone does not inhibit CYP1A expression, nor does it result in a PAHs-resistant phenotype.

Major lipid classes such as triglycerides gradually decrease in embryos for energy metabolism during embryogenesis [92], and the utilization of the lipids could be associated with mobilization of lipid-bound PAHs originally deposited maternally. Reductions in total body lipid and triglycerides were noted in juvenile Chinook salmon fed a PAH-contaminated diet [93]. Three genes involved in lipid binding and transport (StAR related lipid transfer protein 13, FABP H6 isoform, and LDLR 1 precursor) are upregulated in PC embryos, while resistant embryos downregulate FABP H6 isoform and LDLR 1 precursor. Embryos with higher fatty acid metabolism rates likely mobilize bound PAHs at the higher rate, thus increasing PAH embryotoxicity, which could explain the expression pattern of CYP1A among PC embryos. This pattern also indicates that the contribution of paternal PAH-resistant genotype is essential for restricting PAH lipid-bound metabolism in PP embryos. This pattern, coupled with inactivation of CYP1A resulting in lack of PAH activation and formation of toxic PAH secondary PAH-metabolites, may be relevant to adaptive mechanisms among resistant *F. heteroclitus* populations.

It is unclear how much of toxicity is due to a direct maternal deposition vs. maternal gene expression regulation during early developmental stages. Notably, we reported 21% lower average dry weight and downregulation of CYP1A gene among resistant Elizabeth River embryos compared to reference embryos upon exposure to chemically characterized PAH-contaminated sediment extracts [7]. Higher heart rates, the overall smaller size and fatty acid content, and downregulation of CYP1A preventing accumulation of secondary, more toxic PAH-intermediates [81, 90], are likely indicators of adaptive cost among embryos of parents from polluted sites. Additive effect of direct embryos exposure and PAH maternal deposition, evident as a significant increase in embryo PAH-content among resistant embryos exposed to polluted sediment extracts [7], contributes to the overall adaptive cost among resistant embryos. Similar resistance phenotypes were reported among F1 Elizabeth River killifish: exposure to complex PAH mixtures resulted in decreased incidences of cardiovascular deformities, recalcitrant CYP1A expression, elevated basal oxygen consumption rates in embryos, and significantly lower maximal metabolic rates and aerobic scopes among juveniles, compared to King’s Creek killifish [34]. These results, including models integrating genomic and physiological data suggest that toxicity resistance comes at the expense of compromised energetic metabolism, altered mitochondrial function, reduced thermal plasticity, and vulnerability to other environmental stressors [56, 94].

Protein ubiquitination, an important mechanism for cellular protein degradation, is affected by in vitro PAH-exposure. The result is p21 protein degradation [95], causing inhibition of tumor suppression. Moreover, PAH exposure of human HCT116 colorectal carcinoma cell line causes p53-dependent upregulation of CYP1A and activation of detoxification mechanism [96]. Ubiquitin conjugating enzyme E2 17 kDa 3 is upregulated only in PC embryos, which is also evident in the expression patterns of previously discussed CYP1A and several other genes, pointing to PAH-induced p21 ubiquitination and the effect of polluted site maternal deposition.

The metabolic cost to adaptation should be reflected in gene expression governing cellular respiration pathways leading to ATP synthesis and utilization. Eighteen genes (four glycolytic, two TCA cycle, one lactic acid fermentation, one ethanol fermentation, six ETC, one ATP-synthesis, one ATP transportation, and two ATP utilization) are differentially expressed between four embryo families. Patterns of gene expression suggest that the transition phase from glycolysis to the TCA cycle (synthesis of pyruvate to formation of acetyl coenzyme A) is affected. Both sensitive, lower-dosed embryos with diluted sediment extracts (Fig. 3b), and PC and PP embryos developing in clean water (Fig. 4b) displayed elevated heart rates relative to reference embryos in clean water. The upregulation of Na^+^/K^+^-transporting ATPase alpha-3 chain may be relevant to higher heart rates among PP embryos since low molecular weight PAH-induced, AhR-independent cardiac embryotoxicity due to disrupted cardiomyocyte repolarization via K^+^ channel blockade has been reported among sensitive killifish populations [54]. The upregulation of Na^+^/K^+^-transporting ATPase could be another adaptative signature of PAH-resistance in the PP embryos to support their more robust cardiac response to chronic PAH pollution exposure.

PC embryos exposed to PAHs via maternal PAH deposition downregulate PKM, a gene encoding a rate-limiting enzyme during glycolysis [97]. Additionally, downregulation of two genes in the glycolysis-TCA cycle transition step (LDHB and ALDH class 2 mitochondrial precursor), and upregulation of CYP1A suggest a PAH-induced metabolic compromise. Meanwhile, a downregulation of cell-proliferating genes would normally inhibit uncontrolled cell growth and cancer phenotype [98].

PKM and LDHB are directly linked to synthesis and fate of pyruvate leading to either transition from glycolysis to the TCA cycle under aerobic condition or Cori cycle under hypoxia [18]. Since both genes show strikingly opposite patterns—upregulation in CP embryos and downregulation in PC embryos relative to sensitive and resistant embryos—their regulation may be parentally controlled. In the ETC, three NADH ubiquinone oxidoreductases have similar expression patterns, indicating shared regulation, while COX VIIa liver/heart mitochondrial precursor has the opposite patterns of COX VIa precursor and COX subunit VIIIb, suggesting compensating effects.

Selective pressure of a polluted environment can result in the reduction of effective population size by inducing somatic and heritable mutations, population bottlenecks, and non-genetic modes of toxicity. Such effects can ultimately lead to changes in genetic variability and allele frequencies within exposed populations. If there is no selective pressure in terms of pollution on sensitive embryos, the variation in expression levels should be significantly higher when compared to resistant embryos, while variation within crossed groups should fall somewhere between these two. Overall, CC embryos had the highest number of variable genes, as expected. The variation in expression among 114 metabolic genes was significantly lower in PP embryos, relative to sensitive embryos, suggesting that the selective pressure reduces the variation in gene expression among resistant embryos. Both CP and PC embryos show significantly lower numbers of variable genes compared to CC embryos. Interestingly, the PC group has a lower number of variable genes when compared to other groups, suggesting maternal effect on reduced gene expression variability.

## Conclusions

Site chemistry, in vivo morphology, and cardiac physiology results indicate that the PAH-contaminated sediments are toxic to embryos of parents from reference sites, while embryos of parents from polluted sites are remarkably resistant. We report significant heart rate differences during late organogenesis and pre-hatching stages among embryo families from parental crosses of fish inhabiting relatively clean and polluted estuaries. Heart rate differences among sensitive, resistant, and crossed embryos is a reliable phenotype for further explorations of adaptive mechanisms, and metabolic gene expression patterns among embryo families suggest maternal pollutant deposition in the eggs and parental effects on several differentially expressed genes. The observed patterns of 32 significantly altered metabolic genes during late embryogenesis do not reveal a definitive adaptive signature and metabolic cost of resistant phenotypes. Although gene expression patterns among embryo families are suggestive of both maternal and paternal regulation, our results show unexpected sensitive-resistant crossed embryo expression profiles and highlight the complexity of gene-environment interactions in natural populations exposed to pollution. Utilizing next-generation sequencing to differentiate transcriptome response during a critical developmental stage based on chemically characterized pollutant mixtures of sensitive and resistant population crosses may help better understand mechanisms of sensitivity and resistance to polluted environments during animal development.

## Methods

### Sampling sites and *F. heteroclitus* populations

Adult *F. heteroclitus* were captured from three reference and two polluted Superfund sites (Fig. 1). Reference sites are King’s Creek, VA (37°17′ 52.4″ N, 76°25′ 31.4″ W), Magotha Bay, VA (37° 10.6′ N 75° 56.5′ W), and Tuckerton, NJ (39° 32.2′ N 74° 19.4′ W). Polluted sites are Elizabeth River, VA (36° 48.5′ N 76° 17.7′ W) and Newark Bay, NJ (40° 41.2′ N 74° 6.7′ W). Fish were captured by wire mesh minnow traps and transported under controlled temperature and aeration conditions to the NCSU Aquatic Laboratory. Fish were acclimated to common conditions of 20 °C [5, 7, 15, 99] and 15 ppt salinity in 40-gal flow-through re-circulating aquatic system tanks for 4 months prior to laboratory spawning. Effluent from the holding tanks was passed through an activated charcoal filter system and 20% water was changed weekly. Tanks were maintained, and fish were fed (brine shrimp flake, blood meal flake, and Spirulina flake—FOD, Aquatic Biosystems) daily and monitored for health. Fish were maintained under a pseudo-summer cycle (8 h dark/16 h light). Fieldwork and fish collection were completed within publicly available lands. No permission was required for sampling sites access or to collect fish for non-commercial purposes, as *F. heteroclitus* does not have endangered or protected status and does not require collecting permits.

### Reference and polluted sites: chemical analysis

Polluted sediments and water were collected at the southern branch of the Elizabeth River during the incoming tide. The most toxic sediment layer, 5 cm deep, was scraped off the sediment surface, placed in 1 L glass jars, and transported to the lab within 24 h. The multiple sediment sub-samples from each site were combined in the laboratory, thoroughly mixed manually, and placed in stainless steel containers. Sediments were maintained at − 20 °C before extraction. Collected site water was transported in 1 L glass jars without head space and maintained at 4 °C prior to extraction.

Sediments were thawed, mixed with site water at a ratio of 1:1 and shaken for 24 h in 1 L glass jars. The contents were settled for 6 h, and the overlying water was transferred to 250 ml centrifuge bottles and spun at 1000×*g*. The supernatant was transferred to 50 ml centrifuge tubes and spun again at 1000×*g*. The supernatant was serially vacuum-filtered using 3, 1, 0.8, and 0.45 μl Whatman UniPrep filter (PFTE), and the extract was stored in 50 ml polypropylene centrifuge tubes at − 20 °C prior to embryo exposures. Passive sampling devices (PSDs) were deployed in aluminum cages at the following sites: Barnstable, MA, Point Judith, RI, Clinton, CT, Tuckerton Salt Marsh, NJ, Magotha Bay, VA, Manteo, NC, New Bedford Harbor, MA, Newark Bay, NJ, Elizabeth River, VA (Fig. 1 Sites 1–5, 7–10; Table 1). After 14 days, the PSDs were wrapped in combusted aluminum foil and kept on ice. PSDs were then transported to the laboratory and stored at − 20 °C until analysis. PSDs were analyzed using established methods [73, 74].

### Embryo culturing

Eggs and sperm were collected from all five populations of adult fish maintained in the laboratory for 4 months to minimize physiological effect of site-specific exposures *F. heteroclitus* embryos were obtained by in vitro fertilization of individual female oocytes mixed with individual male milt. Developing embryos were examined 24 h post fertilization (hpf) for viability and placed individually into 20 ml glass scintillation vials with 10 ml of treatment solution. King’s Creek embryos were generated by pooling eggs from four females and fertilizing them with four pooled male sperm. Randomly selected embryos per treatment (n = 20) were used for undiluted and serially diluted sediment extract exposures using Elizabeth River contaminated sediments and assessed for morphological deformities, developmental delays, and heart rates.

Eggs from five females from Elizabeth River, VA and Magotha Bay, VA each were fertilized by sperm collected from five males from Elizabeth River and Magotha Bay each resulting in five “clean” (CC) and five “polluted” (PP) family sets with multiple embryo offspring (Additional file 1: Fig. S1). Eggs from “polluted” females were also fertilized with sperm of males from the reference site, and vice versa, yielding crossed embryos (Additional file 1: Fig. S1; clean egg × polluted sperm = CP embryo; polluted eggs × clean sperm = PC embryo). To minimize treatment variation, Magotha Bay and Elizabeth River embryos (including crosses) were reared in 15 ppt filtered seawater at 25 °C and 16 h-light: 8 h-dark cycle and assessed for mortality, developmental delays, morphology, and heart rates at late organogenesis (stage 31) and pre-hatching (stage 35), and gene expression at late organogenesis (stage 31). The Newark Bay and Tuckerton populations were utilized in the same manner to generate sensitive, resistant, and crossed embryos assessed for mortality, developmental delays, and cardiac physiology during stages 31 and 35. Upon reaching a particular developmental stage, surviving embryos were collected as pools of 10–20 embryos from each treatment group, placed in the pre-chilled 1.5 ml Eppendorf tubes, snap-frozen and stored at − 80 °C until used for RNA isolation. Adult fish continued to be housed at 15 ppt salinity in 40-gal flow-through re-circulating aquatic system tanks for use in further research. Experimental procedures for this study, including non-surgical tissue sampling and fish embryo culturing and maintenance were approved by the Institutional Animal Care and Use Committee (IACUC) at North Carolina State University (IACUC assurance A3331-01) and Duke University (IACUC assurance A3195-01).

### Sensitive embryos exposure to polluted sediment extract

Embryos of parents caught at the King’s Creek reference site (CC embryos) were exposed post-fertilization to Elizabeth River contaminated sediment extract supernatant in a dose–response manner. Upon fertilization and confirmation of the successful 4-cell developmental stage, twenty embryos per treatment were placed individually in 20 ml scintillation vials consisting of undiluted and 1:20, 1:10,1:5 diluted sediment extract in a total volume of 15 ml. Vials were placed in an environmental chamber (818 Low Temperature Illuminated Incubator, Precision Scientific, USA) at 20 °C and under 16 h-light: 8 h-dark cycle. Embryos were assessed twice daily for mortality, developmental stage progression, anatomical abnormalities, and cardiac physiology; heart rate were assessed at stages 31 and 35.

### Embryo survival and developmental delays

Developing sensitive/“clean” (CC), resistant/“polluted” (PP) and crossed (CP and PC) embryos were cultured in an environmental chamber (818 Low Temperature Illuminated Incubator, Precision Scientific, USA) at 25 °C, which is within the normal range for *F. heteroclitus* embryo development [99, 100] and the basis for stage-specific gene expression profiling for *F. heteroclitus* embryogenesis, including the late embryogenesis stage 31 and pre-hatching stage 35 [5, 7, 15] and under 16 h light/8 h dark cycle. Fertilization success and embryo progress were monitored twice daily by examining representative stages [15, 16] using a dissecting stereo microscope (Nikon SME1500, Japan), and the time to stage, normal vs. abnormal development, and mortality were noted. Unfertilized eggs, malformed and/or dead embryos were removed from the population, and mortality, times and stages of arrest and abnormal development were recorded accordingly. Survival rates were measured within a family of each population and as overall survival rates between populations. Embryos that successfully reached the 2-cell stage (Stage 3) [15] within a predetermined time were used in the sediment extract exposure experiment. For sediment extract exposures, embryos that successfully hatched and survived to stage 40 as free-swimming larvae were considered survivors.

Criteria for developmental delays was based on > 80% success rate of reference embryos cultured in the vials with 15 ppt filtered seawater and established developmental timelines [15]. Identification of each stage was determined by scoring embryos at predetermined time-periods for both stages 31 (140 h post-fertilization) and 35 (212 h post-fertilization). Once the embryos reached the late organogenesis stage (stage 31) [15, 16], and heart rates and time-to-stage measurement were noted.

### Embryo morphology

At 140 h post-fertilization, randomly selected embryos (n = 20) from each treatment were randomly selected and subjectively scored treatment-blind twice independently (n = 2) for morphological abnormalities using light microscopy. Embryos were scored for severity of heart deformities (tube heart), pericardial edema, hemorrhaging, cranio-facial alterations, tail shortening, and pigment loss. Embryo score was based on a 1–5 scale, 1 representing no deformities, 2-mild, 3-moderate, 4-severe, and 5-extreme, respectively. Non-deformed embryos appeared wrapped approximately 2/3 around the full circumference of the remaining yolk, and with clearly distinguishable cranial ridges, well-defined dark-pigmented eyes with visible retinas, dark and scattered body pigment, clearly distinct atrial and ventricular cardiac regions, absence of hemorrhaging, and the caudal region approximately 1/3 of the body length beginning at the bilobed urinary bladder [3, 15]. Mildly deformed embryos showed minor loss of cranial ridges, slightly smaller and less-pigmented eyes, a more congregated body pigment distribution, and initial hallmarks of cardiac edema, while heart structural integrity was mostly intact. All the markers were more pronounced in moderately deformed embryos, with gradual loss of body pigmentation, increased heart edema, slight tail hemorrhaging and altered tail morphology including overall shortening and widening relative to normally developing embryos; severely deformed embryos had a complete loss of cranial ridges, significantly reduced eye distance, pronounced loss of pigmentation, an increased hemorrhaged membranous area around the heart indicating severe edema and a progressive loss of cardiac integrity; still functioning yet significantly less globular heart lacked the forceful atrio-vertical rhythm, and the blood flow was significantly slower relative to moderately-deformed embryos. The extremely deformed embryos were characterized by smaller size, disproportional size reduction of cranium including diminished distance between eyes, partial-to-complete loss of cranial ridges, reduced eye pigmentation, aggregation and/or reduced body pigmentation, hemorrhaging along the shortened caudal region, and complete loss of cardiac muscle structural integrity characterized by the absence of heart chambers and formation of a thin-walled, translucent “tube heart”. The heart deformities were the most relevant endpoint for determining deformity index scores. Multiple embryo images were recorded by Micropublisher 5.0 RTV Camera (QImaging) fitted on the stereo microscope (Nikon SME1500, Japan) at 70–80× magnification. Embryo images were cataloged, stored, and analyzed using QCapture Pro imaging software.

### Embryo heart rate

Embryo heart rates were determined during early organogenesis and pre-hatching stages (31 and 35, respectively). Individual embryos were tracked to ensure the heart rate was measured from the same embryo at each stage. Each embryo was staged according to established guidelines [16], and embryo images were recorded and cataloged. Embryos were placed in a depression slide under a dissecting stereo microscope for one minute prior to taking heart rate measurements so that the stressed embryo could reestablish a resting heart rate (most *F. heteroclitus* embryos temporarily slow their heart rate due to a sudden change of environment, such as transfer from the Petri dish to a well-lit slide surface). Heart rates of each embryo were established by determining number of beats/30 s (preliminary results showed no significant change in the average heart rate when counts were taken for either 30 s or 1 min). Only embryos that developed successfully to stage 31 and to 35 were considered for analysis. Heart rates of sensitive (CC), crossed (CP and PC) and resistant (PP) embryos from southern (Magotha Bay/Elizabeth River) and northern (Tuckerton/Newark Bay) reference and polluted sites (respectively) developing in clean water were assessed at stages 31 and 35 for population comparisons.

### Embryo gene expression and microarrays

The embryos’ gene-specific mRNA expression was quantified using a *F. heteroclitus* metabolic microarray with four spatially separated replicates per gene on each array. Microarrays were printed using 384 *F. heteroclitus* cDNAs isolated from adult *F. heteroclitus* heart and liver libraries, which include 329 cDNAs that encode essential proteins for cellular metabolism [101]. All expressed sequence tags (ESTs) with enzyme commission numbers or associated with central metabolic pathways from a *F. heteroclitus* EST collection of over 42,000 expressed sequences were included on the array. These cDNAs were amplified with amine-linked primers and printed on 3-D Link Activated slides (Surmodics Inc., Eden Prairie, Minnesota) at the University of Miami core microarray facility.

Individual sensitive (CC), crossed (CP, PC) and resistant (PP) embryos obtained from Magotha Bay (reference) and Elizabeth River (polluted) adult fish population were identified during stage 31 [15, 16] and utilized for RNA isolation, amplification, labeling, and microarray hybridization.

Upon reaching developmental stage 31, embryos were immediately placed in pre-chilled 1.5 ml Eppendorf tubes and snap-frozen at − 80 °C prior to subsequent RNA extraction and microarray analysis. Embryo RNA was extracted using a TRIzol buffer (Invitrogen, Carlsbad, CA, USA) followed by purification using the Qiagen RNeasy Mini Kit (Qiagen Inc., Valencia, CA, USA). RNA for hybridization was prepared by one round of amplification (aRNA) using Ambion’s Amino Allyl MessageAmp aRNA Kit to form copy template RNA by T7 amplification. Amino-allyl UTP was incorporated into targets during T7 transcription; resulting amino-allyl aRNA was coupled to Cy3 and Cy5 dyes (GE Healthcare, Piscataway, NJ, USA).

Labeled aRNA samples (2 pmol dye/µl) were hybridized to slides in 10 µl of hybridization buffer [50% formamide buffer, 5 × SSPE, 1% sodium dodecyl sulfate, 0.2 mg/ml bovine serum albumin, 1 mg/ml denatured salmon sperm DNA (Sigma), and 1 mg/ml RNase free poly(A) RNA (Sigma)] for 44 h at 42 °C. Slides were prepared for hybridization by blocking in 5% ethanolamine, 100 mM Tris pH 7.8, and 0.1% SDS added just before use for 30 min at room temperature, washed for 1 h in 4 × SSC, 0.1% SDS at 50 °C, and then boiled for two minutes in distilled water to denature the cDNAs.

Labeled aRNA samples were hybridized to slides according to the loop design, a randomized incomplete block design that involves constraints of different treatment types so that every comparison is represented by direct contrast on a microarray (Additional file 1: Fig. S1). Each sample was hybridized to two arrays using both Cy3 and Cy5 fluorescent dyes, which is a simple and effective way for the direct comparison of two samples, and for reducing technical variation [102]. As demonstrated in Additional file 1: Fig. S1b, the loop consisted of Cy3 and Cy5 labeled individual embryo aRNA, resulting in a total of 40 replicates (20 biological replicates × 2 dyes for each. Since each array consisted of four replicates of metabolic genes, there were a total of eight gene expression measurements for each embryo (embryo aRNA × 2 dyes × 4 gene replicates = 8 measurements/embryo). The loop formed was CC1-PP1-CP1-PC1-CC2-PP2-CP2-PC2-CC3-PP3-CP3-PC3-CC4-PP4-CP4-PC4-CC5-PP5-CP5-PC5-CC1, where each dash represents a separate hybridization (array) with the individual biological sample at the left of the dash labeled with Cy3 and the biological pool at the right of the dash labeled with Cy5. A number after the treatment (e.g., CC**5**) represents the individual embryo utilized (5th embryo in CC treatment).

Arrays were scanned using a ScanArray Express 4000 (Perkin Elmer). Resulting 16-bit Tiff Images were quantified using ImaGene^®^ (Biodiscovery, Inc.) spotfinding software.

### Data processing and statistical analysis

#### Survival, heart rate, developmental delays, and morphology

Differences in embryo survival, heart rate, and developmental delay among the sensitive, crossed, and resistant populations were analyzed with GraphPad Prism Statistical Software version 9.0.0 (121) using one-way Analysis of Variance (1-way ANOVA, p < 0.05) and Bonferroni multiple comparison test (p < 0.01).

#### Gene expression and microarrays

Not all genes on the array were analyzed, including negative controls (random genomic amplification of Ctenophore specific cDNAs) and genes with either saturated signals or signals with lower intensity than negative controls. The total number of analyzed genes was 179, although most (> 90%) of the metabolic genes expressed during development hybridized to the array based on the signal intensity that was above negative controls.

A linear mixed model was used to test for the difference between sensitive and resistant embryo populations. Log2 measures of gene expression were normalized using linear mixed model in JMP Genomics 9.1 to remove the effect of the dye (fixed effect) and array (random effects), following a spatial Lowess transformation that accounts for signal intensity and spatial bias. Normalized data were modeled by residual maximum likelihood on a gene-by-gene basis using linear mixed model using PROC MIXED. The normalized data was analyzed based on the model Y_ij_ = μ + A_i_ + D_j_ + (A × D)_ij_ + E_ij_, where μ is the sample mean, A_i_ is the effect of the ith array (i = 1–20), D_j_ is the effect of the jth dye (Cy3 or Cy5), (A × D)_ij_ is the array-dye interaction (spot effect), and E_ij_ is stochastic error. Residuals from this model, representing relative “normalized” expression levels, were used for the gene-by-gene analysis of treatment effect (treatment = CC, CP, PC, and PP). This normalized data was modeled using treatment and dye as fixed effects, and array, array-dye interaction, individual, and spot nested in array as random effects, based on the model R_ijkmn_ = μ + A_i_ + D_j_ + (A × D)_ij_ + T_k_ + I_m_ + S(A)_ni_ + E_ijkm_, where T_k_ is the kth treatment (CC, CP, PC, or PP), I_m_ is the mth individual, and S(A)_ni_ is the nth spot nested in the ith array.

For 114 genes significantly differently expressed among individuals (p < 0.01), we examined the variation in gene expression within a family structure on a gene-by-gene basis and compared embryo populations using pairwise comparisons of each family structure via Student’s t-test. If variance in expression for five embryos within a group was larger, the gene was assigned the arbitrary score of one, and the less variable same gene from another population was scored as “zero”. The scores were totaled for 114 analyzed genes and the results are presented as “Percentage of Genes with Higher Variation (%)” between two family structures.

## Supplementary Information

**Additional file 1: Figure S1.** Experimental design for microarray. a Family structure for embryo culturing. “C” represents a parent from Magotha Bay, and “P” represents a parent from Elizabeth River, VA. **b** Microarray design. Each circle represents the aRNA from an individual embryo, and red and green arrows represent Cy3 and Cy5 RNA dye labeling, respectively.

## Data Availability

Microarray data have been deposited in NCBI’s Gene Expression Omnibus and are accessible through GEO Series accession number GSE134501. The map data for Fig. 1 is available under the Open Database License (https://www.openstreetmap.org/copyright).

## Abbreviations

ACC alpha: Acetyl coenzyme A carboxylase alpha
AhR1: Aryl hydrocarbon receptor 1
ALDH class 2 mitochondrial precursor: Aldehyde dehydrogenase, mitochondrial precursor
aRNA: Amplified RNA
cDNA: Copied DNA
COX subunit VIIIb: Cytochrome C oxidase subunit VIIIb
COX VIa precursor: Cytochrome C oxidase polypeptide VIa precursor
COX VIIa liver/heart mitochondrial precursor: Cytochrome C oxidase polypeptide VIIa liver/heart mitochondrial precursor
CYP1A: Cytochrome P450 1A
CYP2P1: Cytochrome P450 2P1
DUSP3: Dual specificity protein phosphatase 3
EST: Expression sequence tag
ETC: Electron transport chain
FABP H6 isoform: Fatty acid binding protein H6 isoform
GMD: GDP-mannose 4,6-dehydratase
HNF 4 alpha: Hepatocyte nuclear factor 4 alpha
STBP1 precursor: Saxitoxin and tetrodotoxin binding protein 1 precursor
LDLR 1 precursor: Low-density lipoprotein receptor 1 precursor
LDHB: Lactate dehydrogenase B chain
Na^+^/K^+^-transporting ATPase alpha-3 chain: Sodium/potassium-transporting ATPase alpha-3 chain
NDU 1 beta subcomplex 6: NADH dehydrogenase (ubiquinone) 1 beta subcomplex 6
NUO B16.6 subunit: NADH ubiquinone oxidoreductase B16.6 subunit
NUO B17 subunit: NADH ubiquinone oxidoreductase B17 subunit
PDHA1 somatic form mitochondrial precursor: Pyruvate dehydrogenase E1 component alpha subunit somatic form mitochondrial precursor
PGK1: Phosphoglycerate kinase 1
PI3K regulatory gamma subunit: Phosphoinositide 3-kinase regulatory gamma subunit
PI3K regulatory subunit polypeptide 3: Phosphoinositide 3-kinase regulatory subunit polypeptide 3 (p55 gamma)
PI4K catalytic beta polypeptide: Phosphatidylinositol 4-kinase catalytic beta polypeptide
PKM: Pyruvate kinase (muscle isozyme)
TCA cycle: Tricarboxylic acid cycle
