# Supplementary figures and images for: Cardiac physiology and metabolic gene expression during late organogenesis among F. heteroclitus embryo families from crosses between pollution-sensitive and -resistant parents
Source: BMC Ecol Evol. 2022 Jan 7;22:3. doi: 10.1186/s12862-022-01959-1 (PMC8739662; doi:10.1186/s12862-022-01959-1)

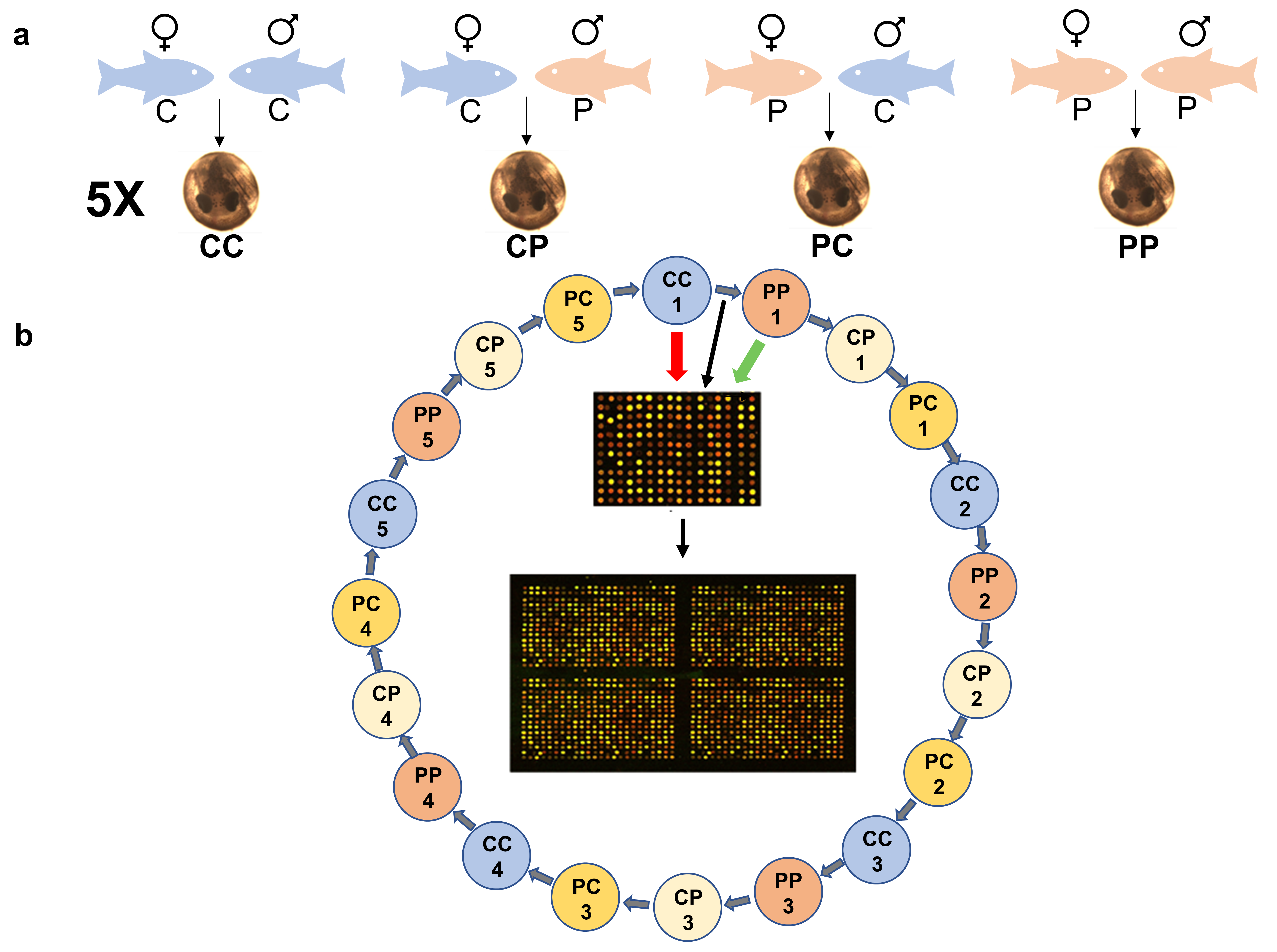

Supplement: Supplementary file 1 — Additional file 1: Figure S1. Experimental design for microarray. a Family structure for embryo culturing. “C” represents a parent from Magotha Bay, and “P” represents a parent from Elizabeth River, VA. b Microarray design. Each circle represents the aRNA from an individual embryo, and red and green arrows represent Cy3 and Cy5 RNA dye labeling, respectively. [file 12862_2022_1959_MOESM1_ESM.tif]
